# Effects of GLP-1 receptor agonists and SGLT-2 inhibitors on cardiac structure and function: a narrative review of clinical evidence

**DOI:** 10.1186/s12933-021-01385-5

**Published:** 2021-09-28

**Authors:** Andrea Natali, Lorenzo Nesti, Domenico Tricò, Ele Ferrannini

**Affiliations:** 1 Department of Clinical and Experimental Medicine, University of Pisa, Via Roma 67, 56100 Pisa, Italy; 2 Present Address: Department of Surgical, Medical and Molecular Pathology and Critical Care Medicine, University of Pisa, Pisa, Italy; 3 CNR Institute of Clinical Physiology, Pisa, Italy

**Keywords:** Type 2 diabetes, SGLT-2 inhibitors, GLP-1 receptor agonists, Heart failure, Treatment, Diuretics, Pathophysiology, Randomized clinical trial

## Abstract

The impressive results of recent clinical trials with glucagon-like peptide-1 receptor agonists (GLP-1Ra) and sodium glucose transporter 2 inhibitors (SGLT-2i) in terms of cardiovascular protection prompted a huge interest in these agents for heart failure (HF) prevention and treatment. While both classes show positive effects on composite cardiovascular endpoints (i.e. 3P MACE), their actions on the cardiac function and structure, as well as on volume regulation, and their impact on HF-related events have not been systematically evaluated and compared. In this narrative review, we summarize and critically interpret the available evidence emerging from clinical studies. While chronic exposure to GLP-1Ra appears to be essentially neutral on both systolic and diastolic function, irrespective of left ventricular ejection fraction (LVEF), a beneficial impact of SGLT-2i is consistently detectable for both systolic and diastolic function parameters in subjects with diabetes with and without HF, with a gradient proportional to the severity of baseline dysfunction. SGLT-2i have a clinically significant impact in terms of HF hospitalization prevention in subjects at high and very high cardiovascular risk both with and without type 2 diabetes (T2D) or HF, while GLP-1Ra have been proven to be safe (and marginally beneficial) in subjects with T2D without HF. We suggest that the role of the kidney is crucial for the effect of SGLT-2i on the clinical outcomes not only because these drugs slow-down the time-dependent decline of kidney function and enhance the response to diuretics, but also because they attenuate the meal-related anti-natriuretic pressure (lowering postprandial hyperglycemia and hyperinsulinemia and preventing proximal sodium reabsorption), which would reduce the individual sensitivity to day-to-day variations in dietary sodium intake.

**Supplementary Information:**

The online version contains supplementary material available at 10.1186/s12933-021-01385-5.

## Introduction

Type 2 diabetes (T2D) is associated with an increased early incidence and severity of heart failure (HF). At a population level, the age-adjusted rate for first hospitalization for heart failure (HHF) in patients with T2D ranges from 4.5 to 9.2 per 1000 person-years, depending on the degree of glycemic control, which is 2–4 times higher than the general population [1]. In real-life care, the age-adjusted incidence of any HHF in T2D is 31 per 1000 person-years, i.e., 2.5-fold (95% CI 2.3–2.7) higher than in well-matched subjects without T2D. Irrespective of the left ventricular ejection fraction (LVEF) value—either preserved (HFpEF: LVEF > 50%), mildly reduced (HFmrEF: LVEF 40–50%), or reduced (HFrEF: LVEF < 40%) [2]— the presence of T2D in chronic HF is associated with a worse prognosis, namely, increased rates of all cause death (11.6 vs. 7.3%/yr), cardiovascular death (9.4 vs. 5.8%/y), all cause hospitalization (47.3 vs. 32.7%/yr), and HHF (13.9 vs. 7.8%/yr). This appears to be somewhat greater in patients with HFpEF (adjusted HHR OR: 2.04 [1.68–2.47]) than in those with HFrEF (adjusted OR: 1.64 [1.44–1.86]).

The observation of increased HF with thiazolidinediones prompted their withdrawal from the market, and boosted cardiovascular safety trials of newer agents, which led, quite unexpectedly, to the discovery of unprecedented cardiovascular benefits of new drugs (and even old ones [3, 4]), especially for patients with HF. Two new classes of drugs might change the natural trajectory of the “lethal synergy” of T2D and HF, namely, glucagon-like peptide 1 receptor agonists (GLP-1Ra) and sodium–glucose cotransporter 2 inhibitors (SGLT-2i). While both classes show comparable benefits on composite cardiovascular endpoints, their effect on the cardiovascular system and their impact on HF-related events appear rather different, and a direct comparison of their efficacy on cardiac structure and function is currently lacking, possibly leaving decision-making in clinical practice difficult.

In this narrative review, we summarize and critically interpret the available evidence emerging from clinical studies focusing on the effects of GLP-1Ra and SGLT-2i on cardiac structure and function, volume homeostasis, and HF-related outcomes. Data from experimental studies, recently and extensively reviewed elsewhere [5, 6], will not be discussed.

## Glucagon like peptide receptor agonists (GLP-1Ra)

### Acute and short-term clinical studies

Beyond its metabolic effects, GLP-1—an endogenous hormone secreted by intestinal endocrine cells—shows interesting direct effects on the myocardium. In human non-failing hearts, GLP-1R is expressed in cardiomyocytes of both atria and, to a negligible extent, ventricles [7]. Acute GLP-1 infusion in humans produces a 40% increase in myocardial microvascular blood flow, both in lean and obese subjects [8]. In a pilot study in 12 patients without T2D and with HFrEF, a 5-week infusion of GLP-1 significantly improved LVEF (from 21 ± 3 to 27 ± 3%, p < 0.01), peak oxygen uptake (VO_2peak_ from 10.8 ± 0.9 to 13.9 ± 0.6 mL/min/kg, p < 0.001), and 6-min walk distance (from 232 ± 15 to 286 ± 12 m, p < 0.001) when compared to standard therapy [9]. This was not confirmed by later placebo-controlled trials in subjects without diabetes with stable HFrEF (FE 30 ± 2%): no amelioration of LV indices was seen after 48-h infusion of GLP-1, while hypoglycemia, tachycardia, and an increase in diastolic blood pressure occurred raising some concerns [10]. A recent meta-analysis of four studies with short term GLP-1 infusion in subjects with HFrEF showed a modest effect on LVEF (+ 4.4%, 95% CI [1.36–7.44]), with no significant change in brain natriuretic peptide (NT-proBNP) levels [11].

GLP-1Ra mimic the “incretin” effect on weight loss and blood pressure (the latter mostly due to afterload reduction and natriuresis [12]); the infusion of exenatide for 6 h in 20 subjects with T2D and decompensated HFrEF induced a small decrease in pulmonary capillary wedge pressure (14.8 to 12.6 mmHg) and an increase in cardiac output from 1.8 to 2.1 L/min, largely due to an increase in heart rate, without affecting NT-proBNP levels [13]. The activation of GLP-1R can provide protection against ischemia–reperfusion injury, probably due to its ability to stimulate myocardial glucose uptake during postischemic contractile dysfunction, although this metabolic shift was never demonstrated in humans [14]. Acute GLP-1 infusion in subjects with acute myocardial infarction both with preserved [14] and reduced LVEF < 40% [15] was associated with an improved LVEF recovery irrespective of diabetic status. In subjects with myocardial infarction and ST segment elevation (STEMI), exenatide administration 15 min before revascularization caused a modest, non-significant reduction in infarct size (dependent on tissue viability and significant [− 30%] in those who received an earlier treatment [16]), with no consequences on left ventricle (LV) function nor clinical events at 30 days [16]. Later, the treatment with either exenatide or liraglutide administered at the time of primary angioplasty in acute myocardial infarction [17, 18] has been consistently proven effective in reducing infarct size by approximately 25% with no effect on LV function, while pre-treatment with *i.v.* GLP-1 was associated with an improved LV dysfunction during elective angioplasty for single-vessel disease [14]. Interestingly, the protective effect on infarct size appears to be time-dependent, so that delayed intervention is associated with no protection [19]. Negative results were also reported by a large, double-blind, placebo-controlled trials on both patients with preserved LVEF receiving exenatide at the time of percutaneous coronary intervention during acute myocardial infarction [20].

Negative results were seen in studies involving exenatide infusion at the time of coronary artery bypass in patients with and without diabetes undergoing elective procedure for coronary artery disease with either reduced [21] or normal LVEF [22]. Interestingly, however, the experimental group experienced lesser arrhythmic events and better hemodynamic stability in the perioperative period. Whether the anti ischemia–reperfusion injury action of GLP-1Ra is also observed in subjects on chronic treatment remains an open question, although a sub analysis of the LEADER study does not support this hypothesis. In the cohort of subjects undergoing myocardial infarction, no difference was observed in the subtype distribution (ST vs. non-ST, symptomatic vs. silent), in death rate or in-hospital troponin levels [23].

### Randomized clinical trials on left ventricular function

A synthesis of the available studies is provided in Table 1. In both a single-arm [24] and a small placebo-controlled RCT [25], chronic treatment with liraglutide in T2D subjects without established cardiovascular disease (CVD) improved diastolic function by reducing filling pressures by 20% (reduced E-wave and E/e′ ratio). This effect is possibly related to the reduction in BMI. On the contrary, no significant effect on diastolic function was observed in two studies comparing liraglutide vs. metformin in newly diagnosed T2D [26] and vs. glimepiride in T2D patients with subclinical HF [27]. Similarly, no difference was seen in two RCTs with GLP-1Ra on HFrEF patients with or without T2D [28, 29]. Liraglutide was essentially neutral on 2D indices of LV systolic function and cardiac output (with a − 3% decline in EF counterbalanced by a slight increase in HR) both in T2D without established CVD [25] and in HFrEF with or without T2D [28, 29]. No significant amelioration of either functional capacity (6-min walk time), LVEF, or natriuretic peptides was evident with other GLP-1Ra on T2D subjects without HF [25] and with HF [30], wherein some concerns about cardiovascular safety (more arrhythmias and ischemic events, possibly secondary to the higher heart rate) were raised in HFrEF [28, 29]. However, in newly diagnosed T2D subjects with subclinical systolic dysfunction as expressed by reduced LV global longitudinal strain (GLS)—an early, less load-dependent systolic index—liraglutide induced an 8% improvement in GLS, which was not entirely justified by the decrease in weight and HbA_1c_ [26].

**Table 1:** Clinical studies on the effects of the therapy with GLP-1Ra on left ventricular function and structure in patients with T2D

Study	Design	Exposure, duration	Outcome, method	Population, n	Baseline	Change (%)	*p*
*Systolic parameters*							
[23]	Prospective vs. placebo	Liraglutide 1.8 μg/die for 26 weeks	LVEF, CMRI	NoHF, 23 vs. 26	55%	− 1%	0.002
[28]	Prospective vs. placebo	Albiglutide various dosage, 12 weeks	LVEF, US	HFrEF, 29 vs. 30	32%	− 2%	ns
[22]	Single arm, prospective	Liraglutide 0.9 μg/die for 26 weeks	LVEF, US	HFpEF, 31	NA	NA	ns
[24]	Prospective vs. aCTRL	Liraglutide 1.8 μg/die vs. metformin 2 g for 6 months	GLS’, US	NoHF, 30 vs. 30	15%	+ 1.2	0.043
[25]	Prospective vs. aCTRL	Liraglutide 1.8 mg, or glimepiride 4 mg for 18 weeks	LVEF, US	NoHF with subclinical dysf, 33 vs. 29	53%	− 2.1	ns
			GLS, US		15%	0	ns
[26]	Prospective vs. aCTRL	Liraglutide 1.8 mg, or glimepiride 4 mg for 25 weeks	LVEF, US	HFrEF, 146 vs. 156	25	+ 1.1	ns
[27]	Prospective vs. placebo	Liraglutide 1.8 mg for 24 weeks	LVEF, US	HFrEF, 122 vs. 119	33%	− 0.7	ns
			GLS, US		11%	0.6	ns
*Diastolic parameters*							
[23]	Prospective vs. placebo	Liraglutide 1.8 μg/die for 26 weeks	E/e′, CMRI	NoHF, 23 vs. 26	7.3	− 0.9	0.001
[22]	Single arm, prospective	Liraglutide 0.9 μg/die for 26 weeks	E/e′, US	HFpEF, 31	12.7	− 2.7	0.0371
[24]	Prospective vs. aCTRL	Liraglutide 1.8 μg/die vs. metformin 2 g for 6 months	E/A, US	NoHF, 30 vs. 30	0.92	+ 0.6	ns
[25]	Prospective vs. aCTRL	Liraglutide 1.8 mg, or glimepiride 4 mg for 18 weeks	E/e′, US	NoHF, 33 vs. 29	12.5	− 0.5	ns
[27]	Prospective vs. placebo	Liraglutide 1.8 mg for 24 weeks	LVEF, US	HFrEF, 122 vs. 119	12.6	− 0.6	0.03
*Remodeling*							
[28]	Prospective vs. placebo	Albiglutide various dosage, 12 weeks	LVDV, US	HFrEF, 29 vs. 30	196	− 0.2%	ns
			LVMi, US				
[23]	Prospective vs. placebo	Liraglutide 1.8 μg/die for 26 weeks	LVEDV, CMRI	NoHF, 23 vs. 26	147	− 11	0.002
			LVMi, CMRI		49	− 1.5	ns
[26]	Prospective vs. aCTRL	Liraglutide 1.8 mg, or placebo 4 mg for 25 weeks	LVEFVi, US	HFrEF, 146 vs. 156	140	+ 6.7	ns
[27]	Prospective vs. placebo	Liraglutide 1.8 mg for 24 weeks	LVEDV, US	HFrEF, 122 vs. 119	163	− 4	ns

In conclusion, whether the activation of GLP-1R signaling is associated with a selective amelioration of LV structure and/or function in subjects with or without ischemic heart disease or overt HF remains unclear. Although GLP-1 increases heart rate and thus cardiac output, evidence for independent effects of GLP-1 on ventricular function is inconclusive [31].

### Randomized clinical trials on heart failure-related outcomes

Although not powered to specifically detect differences in single CV events, all the major CV outcomes trials with GLP-1Ra reported data on HHF. Nevertheless, despite being of different duration and different inclusion criteria, the results altogether provide insight on this outcome. In the LEADER trial [32], T2D subjects with CVD or at high risk for CVD randomized to liraglutide vs. placebo experienced a 13% reduction in the risk of HHF, which was not statistically significant (12 vs. 14 per 1000 person-years; RR: 0.87 [0.73–1.05]). A subsequent analysis showed no difference in HF-related endpoints (hospitalization and CV death) and no safety concerns in patients with HF at baseline [33]. Interestingly, there was no difference in those with or without HF at baseline also in terms of kidney protection (RR 0.77 vs. 0.78) as evaluated by the composite endpoint of new onset microalbuminuria, doubling creatinine and eGFR, renal replacement therapy, and death of renal disease. The SUSTAIN 6 trial [34] recruited a similar population, and despite the positive effects of semaglutide on weight loss (3–4 kg), glycate hemoglobin (HbA_1c_ 0.7–1.0%), renal outcomes (RR 0.64) and MACE (RR 0.74), the rate of HHF was 17.6 vs. 16.1 per 1000 person-years in the semaglutide and placebo arm, respectively (RR 1.11 [0.76–1.61]). No substantial differences were observed related to sex, age, or between patients in primary or secondary CV prevention [35].

**Fig. 1:**
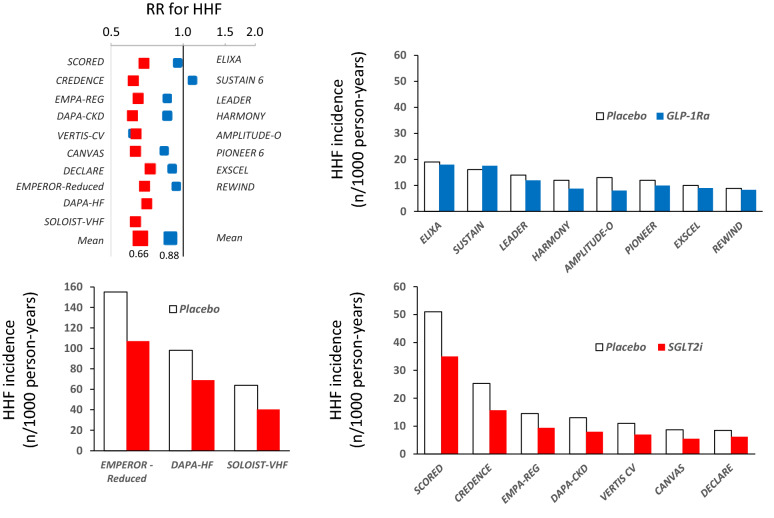
Unadjusted RR and absolute incidence rates of HHF (per 1000 pts-year) in the major CV outcome trials with GLP-1Ra (blue) and SGLT-2i (red)

The population recruited in the ELIXA study [36] was at higher risk of HF, having all participants experienced a recent coronary event and 20% of them a diagnosis of heart failure at entry. Indeed, the incidence of HHF was 19.9 per 1000 person-years, but the treatment with lixisenatide for 24 months did not affect the risk for HHF (RR 0.96 [0.75–1.23]). Similarly, in the HARMONY [37], PIONEER 6 [33], and EXSCEL trials [38], involving T2D subjects at intermediate risk of HHF (10–12 events per 1,000 person-years in the placebo arms), neither albiglutide (RR for CV deaths or HHF 0.85 [0.70–1.04]) nor oral semaglutide (RR 0.86 [0.48–1.55]) nor 1-weekly exenatide (RR 0.94 [0.78–1.13]) significantly reduced the risk of HHF. The REWIND study [39] recruited T2D subjects at a lower risk of HF, being the rate of hospitalization plus urgent visit for HF being 8.9 per 1000 person-years in the placebo arm. Also in this population, the treatment with dulaglutide was not associated with a significant HHF risk reduction (RR 0.93 [0.77–1.12]). The very recent AMPLITUDE-O trial [40] makes an exception since the treatment with efpeglenatide was associated with a 39% reduction in HHF (RR 0.61 [0.38–0.98]). Of note, in terms of MACE and renal outcomes, as well as in terms of HbA1c, blood pressure, heart rate and body weight, the results of AMPLITUDE-O were similar to SUSTAIN 6, LEADER and REWIND trials. Although the population of the study AMPLITUDE-O was relatively more enriched with patients with CVD (90%) and with kidney disease (31%), as shown in Fig. 1, the absolute risk of HHF in the study was similar to other studies and no convincing trend of HHF risk reduction emerges in relation to the absolute risk of the event of the different trials. Why efpeglenatide differs with respect to other GLP-1Ra in terms of HHF prevention remains to be explained.

In conclusion, in individuals with T2D at high-very high CV risk, GLP-1Ra are extremely safe with respect to HF development or decompensation, showing either a neutral or a small beneficial effect. While two metanalyses reported a neutral effect on HHF [41, 42], two other independent meta-analyses [43, 44] estimated a 9% risk reduction of HHF of marginal statistical significance (95% CI 1–17%), which is close to the mean RR of 12% calculated including also the more recent trials (Fig. 1). The data with efpeglenatide are very intriguing and await confirmation, possibly in populations at very high risk of HHF. In cohorts with T2D and HFrEF data is scarce and not encouraging, a statistically non-significant (p = 0.09) 30% increase in HHF was reported in one study [28] and a statistically significant excess of serious adverse CV events (10 vs. 3%) was shown in another report [29], rising possible safety concerns in these more fragile patients.

## Glucose transporter 2 inhibitors (SGLT-2i)

### Acute and short-term clinical studies

SGLT-2i have proved effective in reducing congestion without worsening renal function in acute decompensated HFrEF since the first day of administration [45]. A reduction of cardiac preload can be seen secondary to SGLT-2i induced natriuretic effect, which is in part osmotic (proportional to plasma glucose levels and with a phasic, meal-related pattern) and in part direct from the inhibition of proximal tubular reabsorption of sodium, wherein glucose and sodium are co-transported in a molar ratio of 1:1. Therefore, when SGLT-2 is inhibited, a higher sodium load reaches the distal tubule, translating into a mild (10–20%) increase in sodium excretion which, however, is short lasting (1–3 days) possibly because of some degree of activation of the renin–angiotensin–aldosterone system (RAAS) [46]. The reduction in plasma volume (-9% vs. placebo) appears to be transient, too, as it is clearly detectable at 1 week but not after 12 weeks of treatment [47]. It therefore appears unlikely that the effect of SGLT-2i on heart function is mainly explained by their natriuretic properties alone. Possibly, this effect only emerges in interaction with specific conditions, especially a sequential *nephron blockade* and/or a blockade of the RAAS. In fact, in patients with T2D the natriuretic effect of SGLT-2i was still evident after 5 days only when administered on top of hydrochlorothiazide therapy (25 mg), which by itself had no effect on sodium excretion [48]. This synergistic effect has been also described with loop diuretics: in healthy subjects, when dapagliflozin was given for 7 days after 7 days of bumetanide (1 mg), its first-dose natriuretic effect was twofold greater than when given alone. Moreover, when bumetanide was given after dapagliflozin, its natriuretic effect was 40% greater [49]. This natriuretic effect is coupled with lesser RAAS activation and a more favorable volume redistribution with respect to other diuretics, as has been elegantly demonstrated by the use of bioimpedance spectroscopy [50] as well as modeling data of free water vs. electrolyte-free water clearances [51]. The result is a gentler reduction of plasma volume and a more effective mobilization of interstitial fluids than seen with usual diuretics. Whether in subjects with RAAS blockade or in edematous conditions this effect would be larger and persistent in unknown—but appears plausible. Indeed, a small recent pilot study (EMPA-RESPONSE-AHF trial) [52] has shown that in subjects with or without diabetes presenting with acute heart failure, viz a similar cumulative furosemide dose (320 vs. 300 mg), the initiation of empagliflozin 10 mg was associated with a larger urine output over 4 days (+ 3449, [578–6321] mL) and a more favorable net fluid balance (− 2701, [+ 586 to − 8988] mL) with respect to placebo. Of note, the treatment was continued for 30 days and was associated with a major improvement in 60-days prognosis, being 10 vs. 33% the incidence of the composite outcome (i.e., in-hospital worsening of HF, readmission for HF, and death).

Furthermore, SGLT-2i have been shown to improve endothelial function and reduce arterial stiffness. However, these effects appear modest in size and transient as they are clearly detectable after 2 days of treatment with dapagliflozin [53] but not after 4 weeks [54]. Also, SGLT-2i are known to raise free fatty acids (FFA) and ketone bodies, substrates avidly and efficiently used by the myocardium for energy production [55]. Exogenous β-hydroxybutyrate (β-HB) infusion causes a dramatic 75% increase in myocardial blood flow in normal subjects [56], while in patients with HF it increases cardiac output by 40%, due to an improvement in both stroke volume and heart rate [57]. However, these remarkable effects were observed at plasma β-HB concentrations of 1.6 and 3.3 mM, which are much higher than those achieved in patients on chronic SGLT-2i treatment (0.5–0.8 mM). In addition, these values are reached only in the fasting state, markedly declining in the postabsorptive condition [58]. In the study by Nielsen et al., a more physiologic infusion was tested, yielding a negligible (7%), though statistically significant, increase in cardiac output at a plasma concentration of 0.7 mM [57]. Furthermore, the effect on myocardial blood flow was observed during the simultaneous infusion of insulin, a non-physiological condition.

### Randomized clinical trials on left ventricular function

The available data (Table 2) suggest a significant beneficial effect of SGLT-2i on several variables closely related to cardiovascular outcomes, such as LV systolic and diastolic function as well as LV remodelling. Functional and structural cardiac improvements can be found in T2D patients both with and without overt HF, and across HF subgroups. Yet, a gradient of efficacy and relevance might be detected, with the HFrEF population experiencing greater benefit than the other HF subgroups (HFmrEF and HFpEF), and still lesser changes observed in the non-HF population. In this setting, the effect on LV systolic function expressed as 2D LV EF ranges from 2 to 10% in T2D without HF to 20–30% in T2D with HF either with reduced EF, mildly reduced EF [59] and preserved EF [59–61] show an intermediate 5–10% increase in LV EF. Intriguingly, the improvements in GLS appear to be present across all HF phenotypes with a comparable gradient [59, 62], and to be recognizable despite no increase in LVEF [59]. This pattern is also consistent with the reduction in natriuretic peptides observed with SGLT-2i, more evident in patients with HFrEF [62] and in those with higher baseline peptide values [61, 63]. With regard to diastolic function, there appears to be a more homogeneous 10–15% improvement throughout the different classes of LV EF, more pronounced in subjects with worse baseline values [60].

**Table 2:** Clinical studies on the effects of the therapy with SGLT-2i on left ventricular function and structure in patients with T2D

Study	Design	Exposure, duration	Outcome, method	Population, n	Baseline	Change (%)	*p*
*Systolic parameters*							
[44]	Retr, vs. aCTR	SGLT-2i, 6–24 m	LVEF (%), US	HFp/rEF, SGLT-2i, 74	36.1 (26–48)	+ 8.9 (24%)	< 0.0001
				HFrEF, 45	NA	+ 8.8 (NA)	0.022
				HFmr/pEF, 29	NA	0	ns
				HFp/rEF, NoSGLT-2i, 76	38.8 (28–55)	+ 5.0 (12%)	0.014
				NoHF, SGLT-2i, 78	59.4 (49–64)	+ 2.6 (4%)	< 0.001
				NoHF, NoSGLT-2i, 76	60.4 (52–64)	0	ns
[42]	Retr, vs. DPP4i	SGLT-2i, 2 yrs		CAD, SGLT-2i, 41	46.2 ± 13.5	+ 2.4 (4%)	ns
				HFrEF, 13	29.0 ± 6.2	+ 9.6 (33%)	0.03
				HFmrEF, 7	45.7 ± 3.2	+ 4.3 (9%)	ns
				HFpEF, 21	57.0 ± 4.6	− 1.9 (3%)	ns
				CAD, DDP4i, 40	56.7 ± 16.1	+ 0.4	
[59]	Prosp, vs. aCTR	TOFO 20 mg, 6 m		Outp, SGLT-2i, 21	55 ± 14	+ 5.0 (9%)	0.006
				Outp, NoSGLT-2i, 21	57 ± 18	− 0.6	ns
[60]	Prosp, single arm	CANA 100 mg, 12 m		HFpEF, 35	60.9 ± 1.6	+ 3.7 (6%)	0.023
[61]	1 centre, EMPA-REG	EMPA 10 mg, 3 m		CVD, 10	63 ± 8.0	+ 3.0 (5%)	ns
[62]	Prosp, single arm	CANA 100/300, 3 m		± CVD, 37	65.7 ± 5.0	− 0.4 (1%)	ns
[41]	Prosp, single arm	DAPA 5 mg, 6 m		HFpEF, 53	62.3 (49–68)	+ 1.3 (2%)	0.011
[46]	1 centre, EMPA-REG	EMPA 10 mg, 6 m	LVEF (%), CMRI	CVD, SGLT-2i, 44	58.0 ± 7.5	+ 0.7 (1%)	ns
				CVD, NoSGLT-2i, 46	55.5 ± 8.7	+ 1.0 (2%)	ns
[47]	Prosp, R, vs. aCTR	EMPA 10 mg, 6 m		Outp, SGLT-2i, 20	63.4 ± 1.7	+ 0.2	ns
				Outp., NoSGLT-2i, 8	62.7 ± 2.1	+ 4.2 (7%)	ns
[44]	Retr, vs. aCTR	HF, SGLT-2i, 74	GLS (%)	HFp/rEF, SGLT-2i, 74	− 10.3 (7.3–12.5)	− 1.1 (11%)	0.0001
				HFrEF, 45	NA	− 1.7 (NA)	< 0.001
				HFmr/pEF, 29	NA	− 0.3 (NA)	ns
				HFp/rEF, NoSGLT-2i, 76	− 10.9 (8.4–12.3)	− 0.2 (2%)	ns*
				NoHF, SGLT-2i, 78	− 14.6 (12.1–17.0)	− 0.6 (4%)	0.012
				NoHF, NoSGLT-2i, 76	− 15.2 (12.5–16.9)	0	ns
[41]	Prosp, single arm	DAPA 5 mg, 6 m		HFpEF, 53	15.4 ± 3.4	− 1.4 (9%)	< 0.001
*Diastolic parameters*							
[10]							
[44]	Retr, vs. aCTR	SGLT-2i, 6–24 m	E/e′, US/TD	HF, SGLT-2i, 74	15.6 (11.9–24.3)	− 2.2 (14%)	< 0.001
				HFrEF, 45	NA	− 4.0 (NA)	0.034
				HFmr/pEF, 29	NA	− 1.5 (NA)	ns
				HF, NoSGLT-2i, 76	13.2 (9.8–17.8)	0.0	ns
				NoHF, SGLT-2i, 78	10.6 (9.0–13.5)	0.0	ns
				NoHF, NoSGLT-2i, 76	10.8 (8.9–14.0)	0.0	0.03
[43]	Prosp, single arm	DAPA 5 mg, 6 m		HFpEF, 58	9.3	− 0.8 (9%)	0.02
[42]	Retr, vs. DPP4is	SGLT-2i, 2 yrs		CAD, SGLT-2i, 38	11.4 ± 4.8	− 0.6 (5%)	ns
				CAD, DDP4i, 21	12.9 ± 5.4	− 2.3 (18%)	ns
[41]	Prosp, single arm	DAPA 5 mg, 6 m		HFpEF, 53	9.3 (7.7–11.8)	− 0.8 (9%)	0.020
[59]	Prosp, vs. aCTR	TOFO 20 mg, 6 m		Outp, SGLT-2i, 21	13.0 ± 4.8	− 2.4 (18%)	0.024
				Outp, NoSGLT-2i, 21	13.9 ± 4.6	+ 0.8 (5%)	ns
[60]	Prosp, single arm	CANA 100 mg, 12 m		HFpEF, 35	16	− 6.0 (38%)	< 0.001
[62]	Prosp, single arm	CANA 100/300, 3 m		± CVD, 37	13.7 ± 3.5	− 1.6 (12%)	0.001
[61]	1 centre, EMPA-REG	EMPA 10 mg, 3 m	lateral e′, TD	CVD, 10	8.5 ± 1.6	+ 1.1 (13%)	0.002
*Left ventricular remodeling*							
[44]	Retr, vs. aCTR	SGLT-2i, 6–24 m	LVMi (g/m^2^), US	HFp/rEF, 74	126.3	− 11.1 (9%)	0.026
			LVEDD (mm), US		57.4	− 4.4 (8%)	< 0.01
			LVMi (g/m^2^), US	NoHF, 78	96.6	0.0	ns
			LVEDD (mm), US		49	− 2.0 (4%)	0.036
[43]	Prosp, single arm	DAPA 5 mg, 6 m	LVMi (g/m^2^), US	HFpEF, 58	75.0	− 8.0 (11%)	< 0.001
			LVEDV (mL), US		74.2 (55.1–74.1)	− 5.7 (8%)	ns
[60]	Prosp, single arm	CANA 100 mg, 12 m	LVMi (g/m^2^), US	HFpEF, 35	166.5	− 25.9 (16%)	< 0.001
			LVEDD (mm), US		47.1	− 0.8 (2%)	ns
[61]	Prosp, R, vs. Pl	EMPA 10 mg, 3 m	LVMi (g/m^2^), US	CVD, 10	88	13 (15%)	0.01
			LVEDD (mm), US		47	− 1 (2%)	ns
[47]	Prosp, R, vs. aCTR	EMPA 10 mg, 6 m	LVM (g), CMRI	CVD, 17	93.1 ± 4.8	0	ns
			LVEDV (ml), CMRI		155	− 10 (6%)	< 0.01
[46]	1 centre, EMPA-REG	EMPA 10 mg, 6 m	LVMi, (g/m^2^), CMRI	T2D, 44	59.3 ± 10.9	− 2.6 (4%)	< 0.01
			LVEDV (mL), CMRI		124 ± 33	− 2.9 (2%)	ns

When data were not available, the values were estimated from the graphs

CMRI: cardiac magnetic resonance imaging; CVD: cardiovascular disease; CAD: coronary artery disease; E/e′: mitral E/e′ ratio; US: echocardiography; GLS: global longitudinal strain; HF: heart failure; HFmrEF: heart failure with midrange ejection fraction; HFpEF: heart failure with preserved ejection fraction; HFrEF: heart failure with reduced ejection fraction; LV: left ventricle; LVEDD: left ventricle end diastolic diameter; LVEDVi: left ventricular end diastolic volume index; LVEF: left ventricular ejection fraction; LVMi: left ventricular mass index; ns: not significant; NA: not available

*In the study a p = 0.012 for this comparison is reported, but it is likely a typo

Chronic (6 months) treatment with SGLT-2i is associated with a consistent 5–10% reduction in LV mass index (LVMi). However, it is worth noting that different studies reported discrepant effects on LV linear dimensions and volumes (Table 1), and two trials with the same drug, dose, and duration of treatment recorded opposite results in LVMi and LV end-diastolic volume (LVEDV) as assessed by cardiac magnetic resonance imaging [64, 65]. Hitherto, it is uncertain whether definite LV remodelling occurs with SGLT-2i, at least within a 6-month treatment period.

### Randomized clinical trials on heart failure-related clinical outcomes

Although the CV and kidney outcomes trials with SGLT-2i were not all designed to detect difference in HHF, having also different duration and recruiting subjects with different characteristics, they provide overall a large amount of data and offer interesting insights. An extended analysis of the EMPA-REG OUTCOME trial [66] has provided the following important information regarding the effect of empagliflozin on outcomes: (a) it consistently reduced incident HHF by 30–40%, together with death from HF, investigator-reported HF or edema (as adverse events) and the introduction of loop diuretics, (b) the effect was not influenced by the presence of HF at baseline, sex, race, metabolic control, kidney function, background metabolic and cardiovascular therapy, (c) it was independent of the dose, and (d) it was not associated with more serious adverse events. A similar analysis performed on the CANVAS data [67] extends to canagliflozin most of the above mentioned information related to empagliflozin, apart from the following aspects: the effect on CV death + HHF was more evident in subjects with HF at baseline (RR: 0.61 [0.46–0.80] vs. 0.87 [0.62–1.06], *p* = 0.02 for interaction), in those with body mass index ≥ 30 kg/m^2^, with HbA_1c_ ≥ 8.0%, or on any diuretic. These findings, however, were not confirmed by a preliminary analysis of the CREDENCE trial data [68], which, however, confirmed the efficacy and safety of canagliflozin also in patients with reduced eGFR (44–30 mL/min/1.73 m^2^). Although unable to fully exploit the effect of the drug on glucose control, patients with eGFR 30–60 mL/min/1.73 m^2^ are likely to benefit more from SGLT-2i treatment both in relative (RR: 0.62 [0.51–0.75] vs. 0.71 [0.61–0.82]) and absolute terms (HHF incidence is twofold higher) than those with preserved kidney function. The only study that accurately stratified participants in HF subgroups based on LVEF is the DECLARE-TIMI58 trial [69] showing that the effect of dapagliflozin on reducing the risk of HHF was similar in patients without and with HF, and irrespective of their LVEF value. In contrast, the effect on CV death prevention was particularly evident (almost entirely concentrated) in the subgroup with HFrEF. The DAPA-HF trial in HFrEF demonstrated that dapagliflozin is effective on HHF and cardiovascular death prevention also in subjects without T2D and regardless of either the etiology (ischemic vs. non-ischemic) or the severity of HF (in terms of EF, NT-proBNP levels, and background therapy). In synthesis, as confirmed by a very recent meta-analysis [70], there is very little heterogeneity in terms of HHF prevention among the different subgroups of patients with T2D undergoing treatment with SGLT-2i. Possibly, SGLT-2i could reveal as the only effective treatment in patients with HFpEF [71], wherein several promising drugs proved negative on major endpoints. In fact, the very recent publication of the EMPEROR-Preserved trial has revealed that empagliflozin administration can significantly reduce the number of HHF (HR 0.73; 95% CI 0.61–0.88) in subjects with HFpEF, regardless of the presence or absence of diabetes [72].

**Fig. 2:**
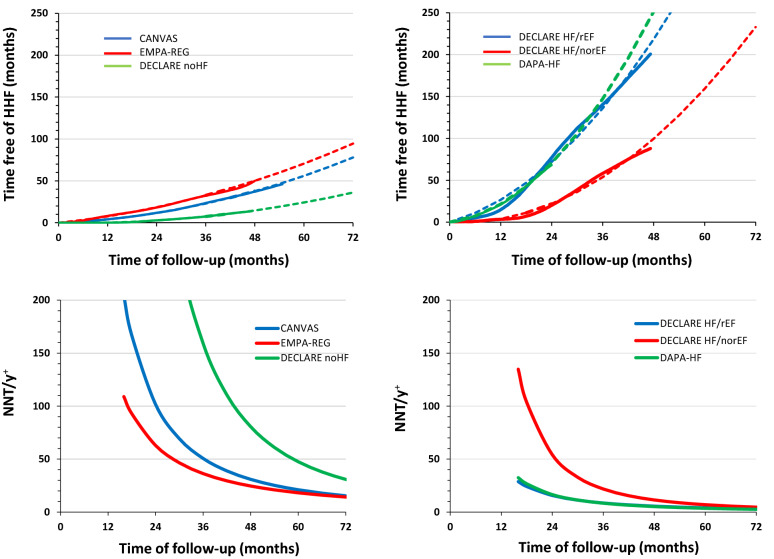
The results of the major CV outcomes trials on patients with SGLT-2i are presented according to the ascending gain in months free of HHF since the beginning of the study (upper panels) for each 100 patients enrolled in the study. The continuous line is calculated from the original Kaplan Majer curves, the dotted line is the best fit calculated from the more robust part of the study (i.e., until at least 50% of the subjects are in the follow-up) then extrapolated to 72 months. In the lower panels data are expressed as NNT that is necessary to gain 1 year free of HHF (NNT/Y^+^) as a function of time

In an attempt to compare the results of major clinical trials with SGLT-2i according to the preexisting heart function of the recruited subjects, we exploited the data of 4 trials for which Kaplan Meier curves of HHF were available and performed the PISA analysis, which provides homogeneous, time-dependent, clinically and economically meaningful estimates of treatment effects in positive clinical trials [73]; a brief description of the method is provided in Additional file 1: Appendix S1. As shown in Fig. 2, the kinetics of the gain in months free of HHF is very different in the cohorts with no or low prevalence of heart failure (EMPA-REG, CANVAS, and DECLARE-noHF: 10, 15 and 0%, respectively) with respect to those with HF (DECLARE, DAPA-HF). In the 3 low-risk cohorts, the difference is largely attributable to the prevalence of cardiovascular disease (EMPA-REG 100%, CANVAS 70% and DECLARE-noHF 36%). In pharmacoeconomic terms, the number needed to treat (NNT) to achieve 1 year free of HHF displays very different kinetics, such that after 3 years of treatment it is 36 NNT in EMPA-REG, 51 in CANVAS, and 159 in DECLARE-noHF; however, the difference attenuates with time. The gain in terms of time free of HHF is dramatically greater in the cohorts with HF (Fig. 2, right panels) showing a gradient according to the baseline impairment in LVEF (DECLARE-HFrEF > DECLARE-HF norEF), but no gradient whether T2D is present in 40% or 100% of the population (DAPA-HF vs. DECLARE rEF cohort). The NNT to achieve 1 year free of HHF after 3 years of treatment is 8.4, 8.5 and 22 in these three cohorts. As expected, from a pharmaco economic perspective, the impact of SGLT-2i on HHF prevention will be in proportion of the individual risk of the event, with a substantial difference also between subjects with preserved or reduced EF. The PISA analysis also indicates that in the long term the treatment is likely to be cost effective in most of the different cohorts.

## SGLT-2i vs. GLP-1Ra: direct comparison and possible mechanisms

The comparison between SGLT-2i and GLP-1Ra can be appreciated by comparing the RR of all randomized clinical trials that included HHF among the outcomes (Fig. 1). Despite the differences across the trials in terms of characteristics of the population, duration and main objective of the study, the data suggest a rather surprising homogeneity in terms of relative risk reduction (− 34% and − 12% with SGLT-2i and GLP-1Ra, respectively. No study has compared SGLT-2i to GLP-1Ra with respect to HF outcomes in a head-to-head design; however, real-world data and meta-analysis estimates are available. A retrospective analysis of real-world data from northern Italy compared the incidence of HHF in 2 large, matched cohorts of T2D subjects during 18 months since the initiation of either class of drugs. SGLT-2i were associated with a reduced rate of HHF (HR 0.59; 95% CI 0.35–0.99; p = 0.048) [74], which is similar in size to what has been reported by the trials comparing SGLT-2i to placebo. Symmetrically, an increased risk of HHF for subjects on GLP-1Ra vs. SGLT-2i emerges from a recent meta-analysis of all available clinical trials (OR: 1.38 95% CI 1.12–1.69; p = 0.002) [75]. This data indirectly confirms the small/neutral effect of GLP-1Ra and the major effect of SGLT-2i in HF prevention and treatment. Improvements in glycaemic control over a 12-month period are known to be associated with an improvement in both systolic (as measured through GLS) and diastolic parameters (expressed by mitral E/e′ ratio) in T2D subjects without overt cardiac disease, irrespective of the glucose-lowering agent used [76]. Despite ameliorated glycemic control, while both GLP-1Ra and SGLT-2i demonstrated reduced atherosclerosis-related events, only the latter showed a significant impact on HF incidence and hospitalization, reflecting the effects on the myocardium that the two drug classes possess. Chronic exposure to GLP-1Ra appears to be essentially neutral on both 2D systolic and diastolic function, irrespective of LV EF; despite this, an amelioration in LV GLS would be possible as well as some degree of protection against ischemic injury. In contrast, a beneficial impact of SGLT-2i is detectable for both systolic (2D LVEF and LV GLS) and diastolic function parameters (E wave, e′, E/e′ ratio) in the T2D population with or without HF, with a gradient reflecting the severity of the baseline dysfunction. An early and significant reduction in LV mass is plausible, while a remodelling of LV dimensions and geometry is still to be proven.

GLP-1Ra and SGLT-2i possess several complementary features concerning their mechanisms of action in patients with T2D; therefore, their combination has the potential to address all the major pathophysiological factors that contribute to the development and progression of T2D and might produce additive cardiovascular benefits. Compared with the single therapy, the combined drugs are able to achieve a higher degree of glycemic control together with a greater body weight loss and lower blood pressure values, despite a comparable incidence of adverse effects [77–83]. Of note, the combination therapy seems to achieve a sustained glycemic control even over long periods of time while being well tolerated and safe [84]. These results have been confirmed in real-world population studies [85, 86] and by metanalyses performed on available data [79, 87]. The dual therapy has demonstrated a greater improvement of endothelial glycocalyx thickness (a marker of endothelial dyfunction) after 12 months of treatment in comparison to insulin therapy, together with a greater reduction in arterial stiffness and a greater increase of myocardial work index, despite a similar improvement of glycemic burden [88]. Based on current knowledge, all these effects are expected to generate an additional CV benefit; however, no study was specifically designed to evaluate CV outcomes, and available metanalyses do not report any additional benefit [42, 89]. Whether the combination of two drugs have some synergism with respect to HF prevention—as it would be expected since both drugs cause natriuresis via different mechanisms—remains to be established with ad hoc trials. In the AMPLITUDE-O trial, 15% of the subjects were on SGLT-2i and the authors state that “the overlap between the RR 95% confidence interval for CV events suggests no interaction”; however, the data on HHF were not specifically commented.

Given the negligible expression of GLP-1R on ventricular cardiomyocytes, the GLP-1R mediated effects on the ventricles are almost certainly indirect and driven by positive modulation of inflammation, endothelial function, and glucose uptake [31, 90]. Furthermore, GLP-1Ra reduce blood pressure in subjects with T2D and hypertension, not strictly dependent on the achieved weight loss [91]; in this setting, sodium handling has been proposed as a GLP-1Ra-mediated effect on cardiac structure, but data are conflicting. In patients with T2D and hypertension, a 3-week treatment with liraglutide elicits a 10% increase in 24-h natriuresis, with a large interindividual variability, without affecting NT-proBNP or blood pressure [12]. In healthy subjects, lixisenatide compared with short-acting insulin increased meal-induced fractional excretion of sodium (absolute change: + 0.25%) but did not affect fasting urinary excretion of other electrolytes or urea. The relative increase in sodium excretion, however, was largely driven by the reduction observed in patients treated with insulin (− 0.14%) [92].

**Fig. 3:**
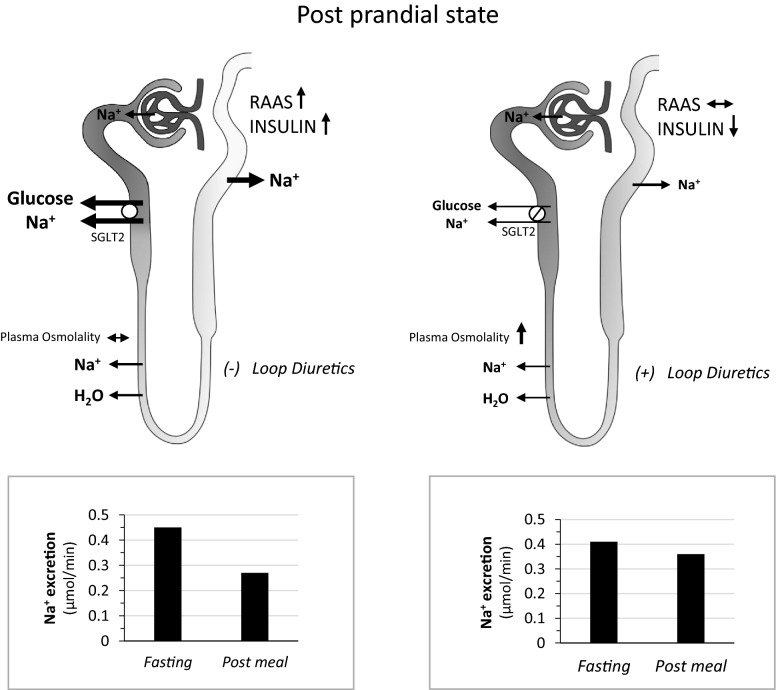
Left panel—in the post prandial state, SGLT-2 is overactive and glucose and sodium are reabsorbed proximally; as a result, distal sodium delivery is reduced and reabsorption is stimulated by a relative activation of the renin–angiotensin–aldosterone system (RAAS) as well as by the physiological rise in insulin levels, which by themselves promote distal sodium reabsorption. The intensity of the grey filling indicates the intraluminal sodium concentration. When sodium concentration is low in the ascending limb of Henle, diuretics are likely to be less effective. Right panel—when SGLT-2 is inhibited, the intraluminal sodium concentration is increased throughout the nephron, less sodium is reabsorbed in the loop because of an increase in osmotic pressure, and less sodium is absorbed in the distal tubule because of lack of RAAS activation and lower insulin levels. When sodium concentration is higher in the ascending limb of Henle, diuretics are likely to be more effective. The histograms at the bottom reproduce sodium excretion rates in fasting and post-meal conditions in T2D subjects before (left) and after (right) SGLT-2 inhibition for 4 weeks (redrawn from Ferrannini et al. [94])

Direct mechanistic effects of SGLT-2i on the myocardium have been suggested [5] as well as effects mediated by the circulating substrate shift [58], but their clinical relevance remains to be established. The effects of SGLT-2i on the kidney are likely to play a major role in mediating the positive effects on HF not only through the preservation of kidney function [93], which probably requires long-term studies to be appreciated, but also through their “smart” diuretic/natriuretic effect. Although the natriuretic effect of SGLT-2i appears small and transient [46], at least in carefully controlled studies, it is plausible that the modulation of the preload emerges in interaction with individual characteristics (background therapy, dietary sodium intake, pre-edematous status) and/or under circumstances that make it more clinically relevant like in the postabsorptive state. In fact, in presence of hyperglycaemia, SGLT-2 is overactive, and more glucose and sodium are reabsorbed proximally; the reduced distal sodium delivery is associated with relative activation of the RAAS, which together with the physiological post-feeding rising in insulin levels promote distal sodium reabsorption by activating the Na/K pump in the distal tubule (Fig. 3). When SGLT-2 is inhibited, the intraluminal sodium concentration is increased throughout the nephron (also because of the lower glucose levels), less sodium is reabsorbed in the loop because of an increase in osmotic pressure, and less sodium is reabsorbed in the distal tubule because of lack of RAAS activation and lower insulin levels. As shown in Fig. 3, the attenuation of the meal-related antinatriuresis (− 15 vs. − 40%) was evident in subjects with type 2 diabetes and normal heart function also after 4-week of treatment with empagliflozin, which of note had no effects on fasting sodium excretion [94]. Through this mechanism, SGLT-2i would make the individuals less sodium-sensitive and their volume regulation less dependent on day-to-day sodium intake variability. In addition, a relatively higher sodium concentration throughout the tubule is likely to make diuretics more effective and also to potentiate the effect of drugs that modulate the RAAS tone. Indeed, T2D and obesity/overeating as well as HF are recognized conditions of relative resistance to diuretics, and empagliflozin in subjects with T2D and chronic, stable HF has been demonstrated to be synergistic with bumetanide in increasing the fractional excretion of sodium also after 14 days of treatment [95] without affecting RAAS tone.

## Limitations and future perspective

We did not use PRISMA guidelines as this manuscript cannot be considered a systematic review. PRISMA guidelines are commonly used for systematic reviews and meta-analysis dealing with outcomes after specific interventions; here, our aim was to condensate and critically evaluate the effects of two classes of drugs on the myocardium, while summarizing their impact on HF-related outcomes and on volume regulation. Therefore, we chose to extensively examine the evidence describing the pathophysiologic bases of the effects of GLP-1Ra and SGLT-2i on heart structure and function focusing only on data in humans (qualitative systematic review [96]).

Despite the lack of clear mechanistic insights, both classes of drugs are widely used in diverse clinical contexts, and clinical guidelines strongly suggest their use especially in patients at higher cardiovascular risk. It will be useful to understand whether it is possible to identify subsets of T2D patients specifically befitting by the cardioprotective actions of one of these two drugs—or of the combination therapy. Both drugs can provide an important and potentially life-saving therapeutic option for many subjects with T2D and known CV benefits: weight loss, blood pressure reduction, and improved glycemic control, while displaying very low risk of hypoglycemia, a known negative prognostic factor in T2D [97]. However, while the treatment with SGLT-2i is associated with a consistent amelioration of cardiac structure and function and HF related outcomes also in HFrEF, in this population the benefit-to-risk ratio for GLP-1Ra remains unknown. Although concerns were not confirmed by a retrospective analysis of several large cohorts of subjects with T2D with and without a pre-existing history of HF under multiple different antidiabetic agents, any relationship between therapy with GLP-1Ra+ and the risk of HHF could be demonstrated [98]. Despite no proven direct effect on heart structure and function and the modest effect on HF-related outcomes in randomized clinical trials, it is possible nonetheless that in the long term the beneficial effects of GLP-1Ra on glycemic control, body weight, blood pressure, as well as on kidney and coronary artery disease may translate into a sizeable benefit in terms of progression to HF or its complications [99].

Currently, while SGLT-2i are recommended as first-line chronic therapy for patients with T2D and HF, either GLP-1Ra or SGLT-2i can be used for the patient at high risk of atherosclerosis-related events [100, 101]. For this latter group of patients, if not achieving target glycemic control, the use of the combination therapy has also been recently suggested [101]. A more pathophysiology driven approach would identify subgroups of patients theoretically benefitting more from the use of these two drugs, either alone or in combination. Obese/overweight, hypertensive subjects at high risk for atherosclerosis-related CV events are the ones expected to benefit more from the therapy with GLP-1Ra. Patients with T2D and cardiac hypertrophy, diastolic or systolic dysfunction or with an hypervolemic state (of either cardiac or renal origin) requiring diuretics, are the best candidate for treatment with SGLT-2i. In this specific setting, a cardiopulmonary exercise test with combined exercise echocardiography might be suggested as an effective strategy for identifying the ones requiring a more aggressive cardioprotective therapy among those with early symptoms of HF (effort intolerance) but no history of coronary events [102, 103]. The combination therapy could be ideal for T2D with a combined phenotype, such as obese with HF of ischemic origin and very high risk of new CV events. Ultimately, the early age of onset of diabetes might also be a criterion for the introduction of GLP-1Ra or SGLT-2i, which have been proven to be the only available disease-modifying drugs for T2D.

## Supplementary Information

**Additional file 1:** A brief description of the method is provided in Additional file 1.

## Data Availability

Not applicable.

## Abbreviations

BMI: Body mass index
CV: Cardiovascular
CVD: Cardiovascular disease
GLP-1Ra: Glucagon-like peptide 1 receptor agonists
HF: Heart failure
HFmrEF: Heart failure with mildly reduced ejection fraction
HFpEF: Heart failure with preserved ejection fraction
HFrEF: Heart failure with reduced ejection fraction
HHF: Hospitalization for heart failure
LV: Left ventricle
LVEF: Left ventricular ejection fraction
LVMi: Left ventricular mass index
NT-proBNP: N-terminal-pro brain natriuretic peptide
NNT: Number needed to treat
RAAS: Renin angiotensin aldosterone system
RCT: Randomized clinical trials
SGLT-2i: Sodium glucose transporter 2 inhibitors
STEMI: ST-elevation myocardial infarction
T2D: Type 2 diabetes
