# Supplementary material for: Effects of GLP-1 receptor agonists and SGLT-2 inhibitors on cardiac structure and function: a narrative review of clinical evidence
Source: Cardiovasc Diabetol. 2021 Sep 28;20:196. doi: 10.1186/s12933-021-01385-5 (PMC8479881; doi:10.1186/s12933-021-01385-5)
Supplement: Supplementary file 1 — Additional file 1: A brief description of the method is provided in Additional file 1. [file 12933_2021_1385_MOESM1_ESM.docx]

**Pragmatic interpretation of survival analysis (PISA) method**

**Background**

Provided that the primary measure of any clinical trial, rather than the event itself, is the time-to-the event, we describe a novel approach to interpret survival analysis data consistent with this premise. Exploiting original Kaplan-Meier survival curves it is possible to generate information with respect to the actual consequences, at the population level, of choosing a treatment *vs* another, which is to provide a gain in event-free life. In addition, by normalizing the time gain of the cohort for its exposure to treatment, time dependent cost-efficacy indices can be generated. This analysis, by generating accurate and meaningful descriptors of the results of any trial reporting the superiority of one treatment/strategy over another, also facilitates the possibility to make comparisons and predictions through time-dependent functions.

**Methods**

*Trials selection.* The characteristics of the clinical trials required to perform an accurate analysis are: a) active treatment *vs* standard or head-to-head comparison, b) superiority clearly demonstrated, c) high quality Kaplan-Meier images, d) outcomes incidence below 50%, e) duration greater than 2 years, f) population size greater than 4,000.

*Data extraction.* As illustrated in **Figure 1**, original inverse KM graphs (*i.e.* indicating cumulative incidence instead of event-free survival) are first captured from the PDF of the article as high definition images (.png) and then converted into data (UN-SCAN-IT Graph Digitalizer software; Silk Scientific, Inc. Orem, Utah USA). Digitalized data (408-1080 couples of time and % values) are visualized and misplaced points (<0.1%) manually shifted. Time (*x* axis) spacing is then forced to 0.25 months yielding sets of 120-266 couples and, if missing, the incidence (*y* axis) value is automatically interpolated with the linear method using the two closest time points. The total variance attributable to the intra- and inter-observer variability was less than 0.1%, ranging between 0.02-0.1%. The accuracy of the data extraction with respect to the original plots can be judged by visual inspection in 3 examples **Figure 2**.

*Data calculation*. The full list of definitions and mathematical formulae is provided in the Glossary (figure 3). The integral function of the inverse KM curves, describing the accumulation of event-free time lost by each treatment cohort is obtained applying piecewise integration using the trapezoid rule. In quantitative terms this function (*Time Lost*) represents the time course of the units of time (months) spent after the specific event has occurred that progressively accumulate in a group of 100 individuals receiving a given treatment during the follow-up. The difference between the integrals of the two treatments, plotted as a function of time, describes the curve of the gain, in terms of event-free time (*Time Gain*) produced by the active treatment. This curve, when the active treatment is effective (i.e. the two original inverse KM curves separates with time), has a peculiar characteristic: in most of the cases it follows a kinetics that can be accurately described by a second order polynomial function forced to pass through the origin (a*time2 + b*time). This was verified also in several CV outcome clinical trials of heterogeneous durations in whom treatments - of different types - were effective like UKPDS-34, STENO-2, CIBIS-II, EMPA-REG OUTCOME, CANVAS, LEADER and SUSTAIN-6 (*Mengozzi A et al. A novel method for interpreting survival analysis data: description and test on three major clinical trials on cardiovascular prevention. Trials 2020, 21(1):578*). The curve fitting for each single outcome in each study under examination is done on data up to the time when 50% of the total population is still being followed-up (Time Gain f50%). This choice was taken to minimize on one hand the uncertainties introduced by a too small number of subjects into the study and on the other, the loss of time-dependent information. The data fit is accepted if regression coefficient values, with respect to the observed curves, were greater than 0.95 and the resulting equation is used to extrapolate the data beyond the time when follow-up was less than 50% and, eventually also beyond the actual duration of the study.

The exposure to the active treatment, expressed as total months of treatment (MoT), is calculated as the integral (area under the curve) of the percent of subjects without the event using the KM *survival* curves derived from the digitalized incidence curves. This time-dependent value is divided for the corresponding time-dependent number of years (i.e. months/12) gained as a consequence of the treatment to generate the curve of the pharmaco economic index MoT/y^+^, which describes how the cost (*i.e.* exposure to the therapy) necessary to gain 1 year of event-free life changes over time during the study. The same procedure is repeated this time using the Time Gain f50% curve instead of observed one. This curve (MoT/y^+^f50%) after the value of 6 months of Time Gain is achieved, follows very closely a power kinetics (a*time^b^) and the equation obtained through the curve fitting (eMoT/y^+^) can be used to estimate the data at fixed time points (eMoT/y^+^@2yrs and eMoT/y^+^@6yrs). The same procedure is repeated replacing the MoT with the actual number of patients being treated at each time point (obtained by dividing the MoT for the time of the study) to obtain the NNT to gain 1 event-free year (NNT/y^+^f50%) and the corresponding eNNT/y^+^@2yrs and eNNT/y^+^@6yrs indices.

**Figure 1 - PISA analysis: flow chart of the steps**


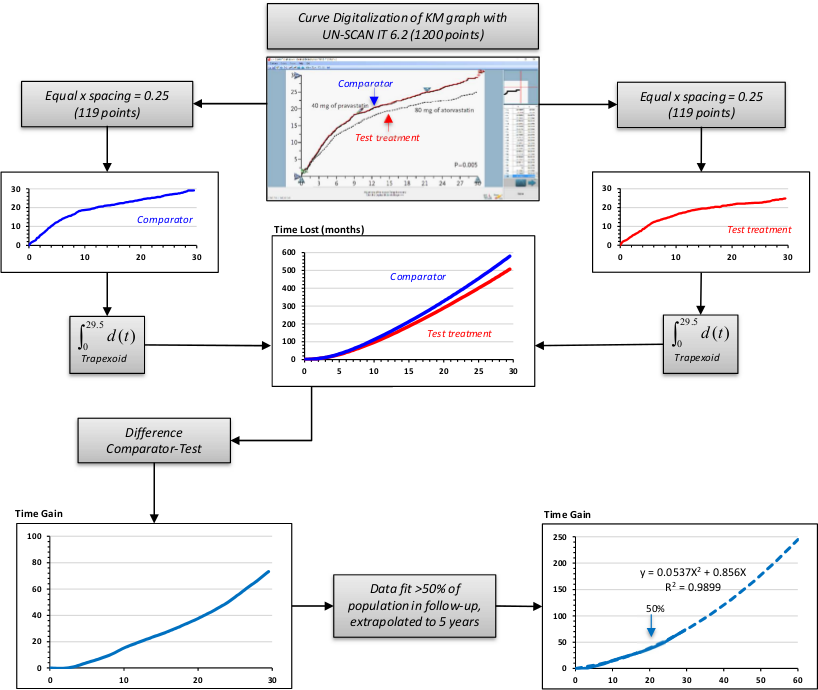


**Figure 2 – Three examples of data extraction and curve fitting**
